# Metabolic engineering of energycane to hyperaccumulate lipids in vegetative biomass

**DOI:** 10.1186/s12896-022-00753-7

**Published:** 2022-08-30

**Authors:** Guangbin Luo, Viet Dang Cao, Baskaran Kannan, Hui Liu, John Shanklin, Fredy Altpeter

**Affiliations:** 1 Plant Molecular and Cellular Biology Program, Agronomy Department, Genetics Institute, University of Florida, IFAS, Gainesville, FL USA; 2 Biology Department, Brookhaven National Laboratory, Upton, NY USA

**Keywords:** *Diacylglycerol acyltransferase*1-2, Energycane, Multigene expression, RNAi, *Oleosin*1, *SUGAR-DEPENDENT*1, Transgenesis, Triacylglycerol, *Trigalactosyl diacylglycerol*1

## Abstract

**Background:**

The metabolic engineering of high-biomass crops for lipid production in their vegetative biomass has recently been proposed as a strategy to elevate energy density and lipid yields for biodiesel production. Energycane and sugarcane are highly polyploid, interspecific hybrids between *Saccharum officinarum* and *Saccharum spontaneum* that differ in the amount of ancestral contribution to their genomes. This results in greater biomass yield and persistence in energycane, which makes it the preferred target crop for biofuel production.

**Results:**

Here, we report on the hyperaccumulation of triacylglycerol (TAG) in energycane following the overexpression of the lipogenic factors *Diacylglycerol acyltransferase*1-2 (*DGAT*1-2) and *Oleosin*1 (*OLE*1) in combination with RNAi suppression of *SUGAR-DEPENDENT*1 (*SDP*1) and *Trigalactosyl diacylglycerol*1 (*TGD*1). TAG accumulated up to 1.52% of leaf dry weight (DW,) a rate that was 30-fold that of non-modified energycane, in addition to almost doubling the total fatty acid content in leaves to 4.42% of its DW. Pearson’s correlation analysis showed that the accumulation of TAG had the highest correlation with the expression level of *ZmDGAT*1-2, followed by the level of RNAi suppression for *SDP*1.

**Conclusions:**

This is the first report on the metabolic engineering of energycane and demonstrates that this resilient, high-biomass crop is an excellent target for the further optimization of the production of lipids from vegetative tissues.

**Supplementary Information:**

The online version contains supplementary material available at 10.1186/s12896-022-00753-7.

## Background

Advanced biofuels are expected to supply 70% of aviation fuel and 50% of the fuel used in freight transport by 2060 [1]. Biofuel enhances the reliability, security, and affordability of the energy supply and reduces carbon emissions to combat global warming [2]. Production of biodiesel or cellulosic ethanol from renewable and perennial feedstocks is expected to result in significant environmental benefit [3]. These fuels are derived from sugars or lipids that are produced in feedstocks via photosynthesis [4–6]. Triacylglycerol (TAG) represents the major lipid component of plant seeds, which provides a highly dense source of energy for the germination and establishment of seedlings [7]. Metabolic engineering of high-biomass crops for the hyperaccumulation of lipids in their vegetative biomass has been proposed as strategy to surpass the oil yields of traditional oilseed crops [8–10].

Under typical growth conditions, plant leaves can synthesize TAG but do not hyperaccumulate it. For example, sugarcane leaves have a TAG content of less than 0.05% of their leaf DW [10]. The biosynthetic pathway for TAG production is highly conserved across species, and the genes encoding the major catalytic steps involved in fatty acid (FA), glycerolipid, TAG production, and hydrolysis have been identified. *WRINKLED*1 (*WRI*1) is a transcription factor and a member of the APETALA2 (AP2)/ethylene-responsive element-binding protein (EREBP) subfamily. It is a positive activator of FA biosynthesis. Overexpression of *WRI*1 has been shown to increase TAG accumulation in *Arabidopsis* leaves 2.8-fold [11]. Suppression of *trigalactosyldiacylglycerol* 1 (*TGD*1) also results in elevation of the extraplastidial FA pool available for TAG assembly and may provide an alternative to the ectopic overexpression of *WRI*1 [12]. TGD1 is a putative component of a lipid transporter that transfers lipids from the endoplasmic reticulum (ER) to chloroplasts [13]. The *Arabidopsis TGD*1 mutant has increased TAG accumulation in leaves [14]. Diacylglycerol acyltransferase1-2 (DGAT1-2) is a rate-limiting enzyme for the conversion of diacylglycerol into TAG [15]. Oleosin1 (OLE1) is a structural protein that protects lipid droplets from coalescence and reduces lipid turnover [16, 17]. A synthetic OLE1 (CysOLE1) with six engineered cysteine residues improves FA contents in vegetative tissues of *Arabidopsis* [18]. After TAG is synthesized in the ER, SUGAR-DEPENDENT1 (SDP1), a specific TAG lipase, can catalyze its hydrolysis [19]. The suppression of *SDP*1 has been shown to increase TAG accumulation in vegetative tissues [20, 21]. Similarly, the mutation of a subunit of the peroxisomal fatty acid ABC transporter (PXA1), which contributes to lipid transport across the peroxisomal membrane for β-oxidation, results in an increase in TAG accumulation in expanding leaves [22].

A “push–pull–protect” strategy has been proposed to increase plant lipid content in vegetative tissue [23]. In this strategy, the genes involved in lipid synthesis (push) and TAG assembly (pull) are overexpressed, while lipid turnover is suppressed (protect) through multi-gene engineering. Indeed, TAG accumulation has been found to increase in vegetative tissues of both model plants such as *Arabidopsis thaliana*, *Brachypodium distachyon*, *Nicotiana benthamiana*, and *Nicotiana tabacum* [23–27] and high biomass crops including sugarcane [10, 28], maize [29], sorghum [30], and perennial ryegrass [31], through the overexpression of *WRI*1, *DGAT*1-2, and *OLE*1 and/or the suppression of *SDP*1 or *PXA*1. For example, in one study, the constitutive coexpression of *WRI*1, *DGAT*1-2, and *CysOLE*1 and simultaneous RNAi-mediated cosuppression of *ADP-glucose pyrophosphorylase* (*AGPase*) and a subunit of *peroxisomal ABC transporter*1 (*PXA*1) elevated TAG accumulation in leaves of transgenic sugarcane to 1.90% of dry weight (DW), compared to 0.02% of DW in non-modified sugarcane [10].

Energycane, like sugarcane, is an interspecific hybrid in the genus *Saccharum*. By contrast to sugarcane, energycane has a high proportion of the ancestral species *Saccharum spontaneum* in its genome, which contributes to higher tiller number, greater biomass yield, more fiber content, and better persistence, in addition to reduced stem diameter and sugar content [32]. Energycane is an ideal feedstock for the production of lipids in vegetative tissues, due to its superior biomass production, tolerance to pests and diseases, persistence on marginal land, and elevated cold tolerance [33–36]. However, it is among the most recalcitrant species in regard to tissue culture and genetic transformation; to date, there have been few reports of successful energycane transformation. Two such reports provided detailed biolistic transformation protocols using *neomycin phosphotransferase* II (*npt*II) [37] or *bar* as a selectable marker gene [38]. A third report described the use of transgenic energycane as a platform for producing a recombinant therapeutic protein via overexpression of a cDNA encoding snowdrop lectin [39]. Here, we report what is to the best of our knowledge the first metabolic engineering of energycane. Following multi-gene engineering for the hyperaccumulation of TAG, the levels of transgene expression/target gene suppression and the TAG and total FA accumulation in leaves of transgenic energycane were analyzed.

## Results

### PCR analysis of transgenic energycane

**Fig. 1:**
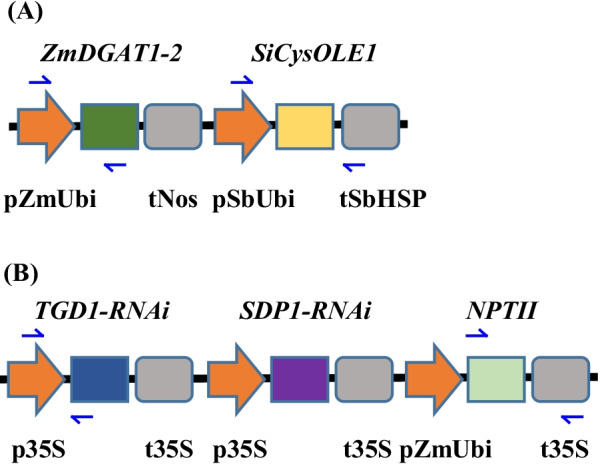
Multi-gene expression and RNAi suppression constructs that were cotransformed into energycane. **A** The overexpression cassette of *ZmDGAT*1-2 and *SiCysOLE*1. **B** RNAi hairpin cassette of *TGD*1 and *SDP*1. Neomycin phosphotransferase II (NPTII) is used as the selectable marker. *ZmDGAT*1-2: *Diacylglycerol acyltransferase1-2* from *Zea mays*. *SiCysOLE*1: *Cysteine oleosin*1 from *Sesamum indicum*. *TGD*1: *Trigalactosyl diacylglycerol*1 from *Saccharum* spp. hybrid. *SDP*1: *Sugar-dependent*1 from *Saccharum* spp. hybrid. pZmUbi and pSbUbi: Ubiquitin promoters from *Zea mays* and *Sorghum bicolor*, respectively. tNos: Nos terminator from *Agrobacterium tumefaciens*. tSbHSP: The heat shock protein 18 terminator from *Sorghum bicolor*. p35S and t35S: 35S promoter and terminator, respectively. Half arrows indicate primers used to detect transgenes

**Fig. 2:**
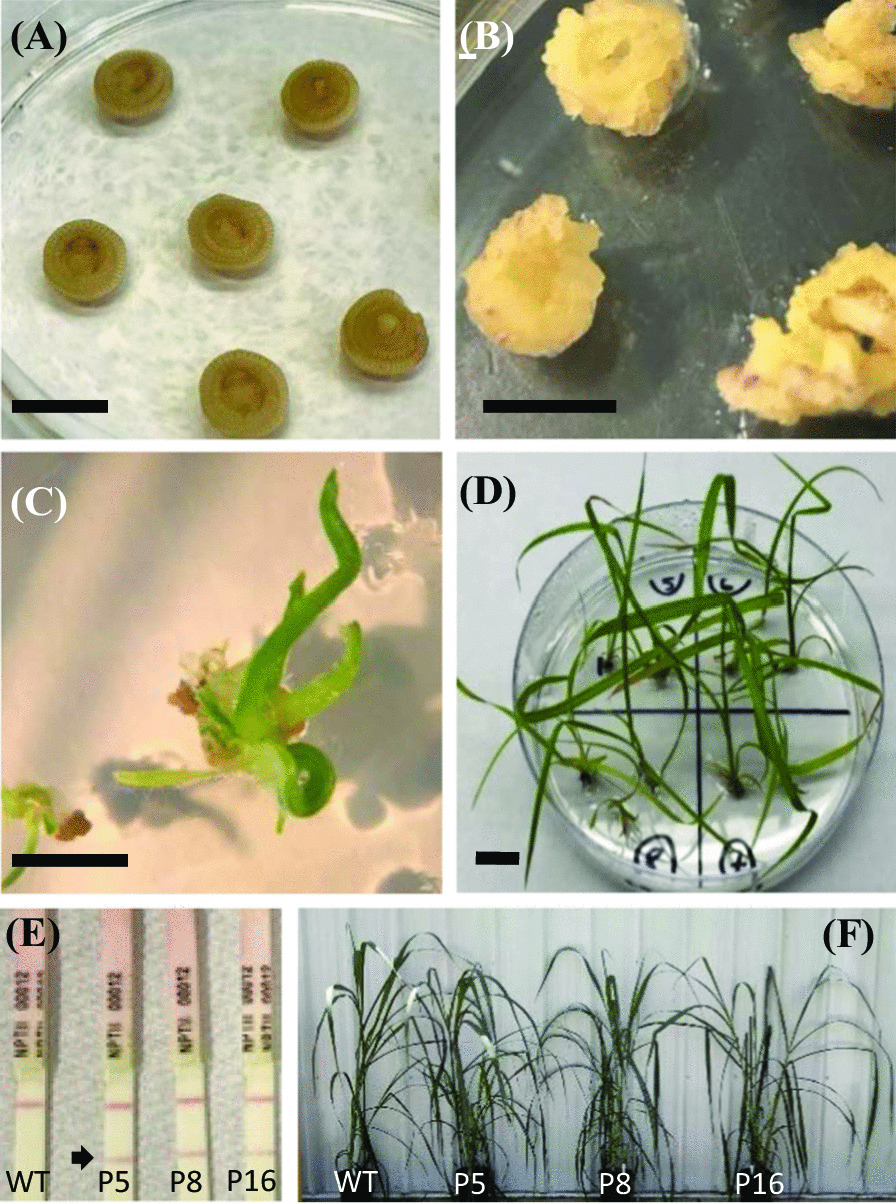
Generation of transgenic energycane. **A** Leaf whorl cross-sections were used as explants.** B** Callus induced from leaf whorl cross-section. **C** Calli regenerating on medium with geneticin for selection. **D** Regenerated plants before transfer to soil. **E** NPTII immuno-chromatography of crude protein extracts from transgenic energycane lines (P5, P8, and P16) and non-transgenic energycane (WT), arrow NPTII test line, Bar = 1 cm. **F** Transgenic energycane lines (P5, P8, and P16) next to non-transgenic energycane (WT)

PCR primer pairs were designed to amplify both the 5′ and 3′ ends of each of the two cotransformed, unlinked recombinant DNA constructs (Fig. 1; Additional file 1: Table S1) containing the overexpression cassettes of *ZmDGAT*1–2 and *SiCysOLE*1 and/or the RNAi suppression cassettes of *SDP*1 and *TGD*1, in addition to the *npt*II selectable marker gene (Fig. 1A, B). Biolistic gene transfer and the regeneration of transgenic plants (Fig. 2) resulted in 31 transgenic lines from 13 shots, as confirmed by NPTII immuno-chromatography and *npt*II PCR (Fig. 2; Additional file 1: Table S2). Of the 31 transgenic lines, eight (P5, P8, P16, P19, P24, P26, P27, and P28) were confirmed by PCR to contain both of the cotransformed linearized, unlinked recombinant DNA constructs carrying the overexpression cassettes of *ZmDGAT*1-2 and *SiCysOLE*1, as well as the RNAi suppression cassettes of *SDP*1 and *TGD*1, in addition to *npt*II (Table 1 and Additional file 1: Table S2 and Fig. S1); these were selected for further quantitative real-time PCR (qRT-PCR) and TAG analyses.

**Table 1:** Summary of TAG content and expression/suppression of transgenes in leaves of transgenic energycane

Line	TAG (% of DW)	Expression/suppression of transgenes in leaves			
		*ZmDGAT*1-2	*SiCysOLE*1	*TGD*1 (%)	*SDP*1 (%)
CP 82-1592	0.05 ± 0.02^a^	0.00 ± 0.00^a^	0.00 ± 0.00^a^	100 ± 19^ab^	100 ± 9^abcd^
P27	0.11 ± 0.05^a^	0.08 ± 0.01^abc^	0.02 ± 0.00^cd^	39 ± 6^ef^	152 ± 37^e^
P24	0.12 ± 0.07^a^	0.00 ± 0.01^a^	0.01 ± 0.00^a^	51 ± 15^de^	119 ± 12^abcd^
P19	0.12 ± 0.01^a^	0.06 ± 0.01^ab^	0.00 ± 0.00^ab^	106 ± 12^a^	120 ± 13^abcde^
P26	0.13 ± 0.00^a^	0.14 ± 0.01^bcd^	0.02 ± 0.00^cd^	33 ± 6^ef^	130 ± 32^cde^
P25	0.18 ± 0.02^a^	0.16 ± 0.01^cd^	0.00 ± 0.00^a^	23 ± 10^f^	149 ± 11^e^
P28	0.19 ± 0.05^a^	0.15 ± 0.02^bcd^	0.03 ± 0.00^cd^	18 ± 2^f^	121 ± 31^bcde^
P20	0.21 ± 0.01^a^	0.20 ± 0.09^cd^	0.03 ± 0.01^cd^	100 ± 16^ab^	139 ± 7^abcde^
P6	0.21 ± 0.05^a^	0.31 ± 0.04^f^	0.02 ± 0.00^cd^	113 ± 3^a^	169 ± 11^de^
P23	0.30 ± 0.18^a^	0.30 ± 0.02^ef^	0.00 ± 0.00^a^	98 ± 17^ab^	174 ± 11^e^
P7	0.36 ± 0.02^a^	0.34 ± 0.01^f^	0.07 ± 0.00^e^	104 ± 17^a^	105 ± 8^abcd^
P5	0.65 ± 0.09^ab^	0.55 ± 0.07^g^	0.26 ± 0.01^f^	65 ± 7^cd^	70 ± 12^af^
P8	0.90 ± 0.06^ab^	1.63 ± 0.06^k^	0.01 ± 0.00^bc^	51 ± 15^de^	95 ± 16^abc^
P16	1.52 ± 0.45^bc^	1.44 ± 0.08^h^	0.01 ± 0.00^ab^	70 ± 1^bcd^	81 ± 16^abf^

TAG and gene expression values shown for each line represent leaf extracts from three biological replicates. Values are expressed as means ± SDs. Values within one column with different letters are significantly different at *p* ≤ 0.05 according to a one-way ANOVA test and the Duncan’s Multiple Range Test (MRT), administered post hoc. The expression of transgenes is shown relative to *GAPDH*. Suppression of RNAi target genes is shown as a percentage of the non-modified CP 82-1592 control

Of the 31 transgenic lines, eight were only PCR-positive for the *npt*II selectable marker and RNAi suppression cassettes. Of the 31 transgenic lines, 15 were PCR-positive only for one of the two PCR reactions that were carried out for each of the two cotransferred recombinant DNA constructs, indicating fragmented inserts. Lines P6, P7, and P20, which were PCR-positive for *ZmDGAT*1-2; *SiCysOLE*1, and line P23, which was PCR-positive for *ZmDGAT*1-2, were also included in the qRT-PCR and TAG analyses (Additional file 1: Table S2). The non-transgenic energycane CP 82-1592 (wild type, WT) was used as negative control. Sugarcane line 1565, described by Parajuli et al. [28], which was grown under the same conditions, was also included in RT-PCR and TAG analyses.

### TAG and total FA accumulation in the leaves of transgenic energycane and sugarcane

**Fig. 3:**
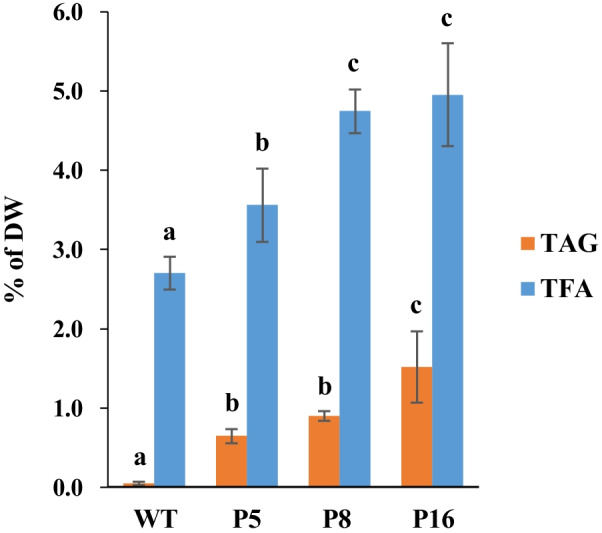
TAG and total FA content of transgenic energycane lines (P5, P8, and P16). Values with different letters are significantly different at *p* ≤ 0.05 according to a one-way ANOVA test and the Duncan’s Multiple Range Test (MRT), administered post hoc. P5, P8, and P16 are lines with the highest TAG content compared to other transgenic lines. *WT* Non-transgenic energy cane, *TAG* Triacylglycerol, *TFA* Total fatty acid, *DW* dry weight

The transgenic lines had a TAG content ranging from 0.11 to 1.52% of leaf DW, which was 2- to 30-fold of that of WT (Table 1). Sugarcane line 1565, described by Parajuli et al. [28], was also grown under the same conditions and displayed TAG contents of 4.87% of leaf DW (Additional file 1: Table S3). The energycane lines P5, P8, and P16, which had the highest TAG contents at 0.65%, 0.90%, and 1.52% of leaf DW (Fig. 3), respectively, were selected to analyze total FA content as well as TAG and total FA composition. P5, P8, and P16 had a total FA content of 3.56%, 4.74%, and 4.96% of leaf DW, respectively, the latter being almost double that in WT (2.70% of DW) (Fig. 3). The total FA content was highly positively correlated with the TAG content (0.93) in Pearson’s correlation analysis (Additional file 1: Table S4).

### TAG and total FA composition in leaves of transgenic energycane

**Fig. 4:**
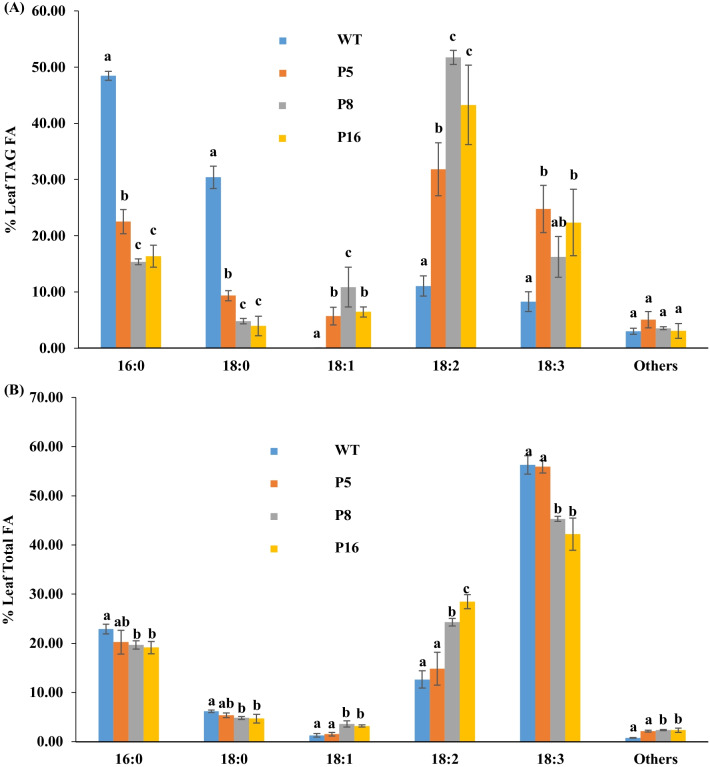
TAG composition and total FA composition in leaves of transgenic energycane lines with higher TAG content. **A** Different TAG compositions in leaves of transgenic lines and non-transgenic energycane (WT). **B** Different total fatty acid compositions in leaves of transgenic lines and WT. Values with different letters are significantly different at *p* ≤ 0.05 according to a one-way ANOVA test and Duncan’s Multiple Range Test (MRT), administered post hoc. P5, P8, and P16 are lines with higher TAG content than other transgenic lines. *TAG* Triacylglycerol, *FA* fatty acid

The composition analysis of TAG FA revealed that the unsaturated FA, oleic acid (C18:1Δ9), increased from 0.00% in WT to 5.68–10.86% leaf TAG FA in transgenic lines P5, P8, and P16. The content of the saturated FAs, palmitic acid (16:0), and stearic acid (18:0) was reduced in the transgenic lines to 12.97–46.52% of those of WT (Fig. 4A). The amount of linoleic acid (LA, 18:2^Δ9,12^) was significantly increased in transgenic lines 2.88- to 4.68-fold, at the expense of α-linoleic acid (ALA, 18:3^Δ9,12,15^) (Fig. 4A). Similarly, in the total FA composition analysis, the content of C18:1Δ9 and 18:2^Δ9,12^ of the three lines was significantly elevated 1.17- to 2.80-fold compared to WT, while the content of 16:0, 18:0, and 18:3^Δ9,12,15^ was reduced to 74.98–88.40% of the WT levels (Fig. 4B). The levels of other FAs in all three lines were increased 1.02- to 3.12-fold compared to those of WT in both the TAG and total FA composition analyses (Fig. 4A, B).

### Overexpression/suppression levels of transgenes in transgenic energycane and sugarcane

qRT-PCR analysis was conducted to examine the levels of transgene expression and target gene suppression in the split leaves used for the TAG and FA analyses. *ZmDGAT*1-2 was overexpressed, with a relative expression level ranging from 0.06 to 1.63, in all transgenic lines, except for line P24, which did not express *ZmDGAT*1-2 (Table 1). *SiCysOLE*1 was expressed at less than 0.1 relative to *glyceraldehyde 3-phosphate dehydrogenase (GAPDH)* in transgenic lines, except for line P5, which had an expression level of 0.26 relative to *GAPDH* (Table 1). *TGD*1 RNAi resulted in a transcript suppression of up to 18% of WT levels (line P28; Table 1). The suppression of *TGD*1 was identified in both lines with high TAG contents ranging between 0.65 and 1.52% TAG of DW (P5, P8, and P16) and lines with low TAG content ranging between 0.11 and 0.19% TAG of DW (P24, P25, P26, P27, and P28) (Table 1). Interestingly, most lines with *ZmDGAT*1-2 and/or *TGD*1 suppression showed a 5–74% elevated *SDP*1 expression relative to WT levels despite the cotransformation with an *SDP*1 RNAi construct. However, the lines that had the highest TAG accumulation (P8, P16, and P5) displayed modest *SDP*1 suppression, with 95%, 81%, and 70% of WT levels, respectively (Table 1). The transgenic lines that had the highest TAG content (P5, P16, and P8) displayed the highest relative expression level of *ZmDGAT*1-2 at 0.55, 1.44, and 1.63, respectively. By contrast, lines with low TAG content ranging between 0.11 and 0.19% TAG of DW, such as P19, P24, P25, P26, P27, and P28, displayed a relative *ZmDGAT*1-2 expression level of less than 0.16 (Table 1). By contrast to energycane lines P5, P8, and P16, sugarcane line 1565 displayed *WRI*1 expression (0.05 relative to *GAPDH*) and stronger suppression of *SDP*1 (30% relative to WT levels), while *ZmDGAT*1-2 (0.19 relative to *GAPDH*) and *SiCysOLE*1 expression (0.02 relative to *GAPDH* Additional file 1: Table S3) were lower than in the highest-expressing energycane lines.

### Correlation between the levels of transgene expression/target gene suppression and TAG accumulation in transgenic energycane

Pearson’s correlation was evaluated for TAG accumulation and the levels of expression of lipid genes in leaves of transgenic lines. As shown in Table 2, TAG accumulation was highly positively correlated with the expression level of *ZmDGAT*1-2, with a correlation coefficient of 0.86 while it was negatively correlated with the expression level of *SDP*1 with a correlation coefficient of − 0.52. However, TAG content was not significantly correlated with the expression of *SiCysOLE*1 or *TGD*1 in transgenic energycane (Table 2).

**Table 2:** Correlation of (trans)gene expression with TAG content in transgenic energycane

	TAG	*ZmDGAT*1-2	*SiCysOLE*1	*TGD*1	*SDP*1
TAG	1.00				
*ZmDGAT*1-2	0.858**	1.00			
*SiCysOLE*1	0.244	0.136	1.00		
*TGD*1	− 0.059	0.003	0.016	1.00	
*SDP*1	− 0.520**	− 0.437**	− 0.362*	− 0.285	1.00

Very significant differences in correlations were detected for TAG with *TGD1* and *SDP1* and significant differences in correlation were detected for TAG with *SiCysOLE1* according to a oneway ANOVA test and the Duncan’s Multiple Range Test (MRT), administered post hoc

*Correlation is significant at the 0.05 level (*p* ≤ 0.05)

**Correlation is very significant at the 0.01 level (*p* ≤ 0.01)

## Discussion

Energycane produces among the highest amounts of biomass of any crop, which, in combination with its resilience and ease of biocontainment, makes it a prime feedstock candidate for fueling the emerging bioeconomy [33–36]. However, its recalcitrance in regard to tissue culture and genetic transformation has so far prevented complex metabolic engineering and molecular pharming approaches. To the best of our knowledge, only one earlier report presents energycane as a production platform for recombinant protein [39].

Here we report the first complex metabolic engineering of energycane, which resulted in a hyperaccumulation of lipids in vegetative biomass. Using a multi-gene approach with constitutive overexpression of transgenes *ZmDGAT*1-2 and *SiCysOLE*1 and the RNAi suppression of *TGD*1 and *SDP*1, transgenic energycane accumulated TAG at 1.52% of leaf DW in an average of three biological replicates. This TAG accumulation exceeds that detected in non-transgenic energycane (WT) by 30-fold (Table 1). The level of TAG hyperaccumulation also resulted in almost twice the total fatty acid content relative to WT (Fig. 3). Similarly, TAG accumulation has been increased through metabolic engineering of lipid biosynthesis genes in vegetative tissues of model plants and crops [10, 23–31]. In perennial ryegrass (*Lolium perenne*), the accumulation of TAG in leaves was increased 14-fold relative to WT to 2.5% of leaf DW when *AtDGAT*1 and *SiCysOLE*1 were coexpressed [31]. Interestingly, TAG accumulation was closely correlated to *SiCysOLE*1 expression and not to *AtDGAT*1 expression in ryegrass, suggesting that TAG degradation may be higher in ryegrass than in energycane. By contrast, in this work, TAG was most highly correlated with *ZmDGAT*1 expression and was not correlated with *SiCysOLE*1 expression. Differences in the source of the *DGAT*1 gene, codon optimization, and promoter choice, which affected the expression of the individual genes, may also have contributed to these contrasting findings.

*ZmDGAT*1-2 was also found to be positively correlated with TAG accumulation in transgenic sugarcane lines coexpressing *SbWRI*1, *ZmDGAT*1-2, and *SiCysOLE*1 and suppressing *AGP*ase and *PXA*1 [10]. While *ZmDAGT*1 was highly expressed in energycane, *SiCysOLE*1 expression was very modest in the majority of lines, and the optimization of its expression has the potential to boost TAG accumulation. The *SDP*1 suppression level was also highly correlated with TAG accumulation in energycane. This finding is in agreement with earlier studies in *Arabidopsis*, where a mutant background in *SDP*1 severely decreased FA turnover, leading to more than a twofold increase in leaf TAG accumulation when stacked with *WRI*1 and *DGAT*1 overexpression [21] or a 15-fold increase in leaf TAG when stacked with *TGD1* suppression [20]. However, *TGD*1 suppression to 18% of WT levels in energycane was associated with only modest TAG accumulation (0.19% of leaf DW) and therefore did not correlate with TAG accumulation (Tables 1 and 2). Stronger levels of target gene suppression may be facilitated by the gene editing approaches that were recently established for sugarcane [40–43], in which the highly polyploid nature of sugarcane and energycane offers the opportunity to create a range of knockout levels and phenotypes.

Gene expression and TAG accumulation were also compared directly between the transgenic energycane lines generated in this work and the transgenic sugarcane line 1565, which was reported previously [28]. Sugarcane line 1565 harbored the same gene expression/RNAi suppression cassettes as energycane in addition to a constitutive expression cassette of *WRI*1. The expression of *WRI*1 and stronger suppression of *SDP*1 in sugarcane line 1565 contributed to a 3.2-fold higher TAG accumulation than in energycane line P16 (Table 1 and Additional file 1: Table S3). A synergism between *WRI*1 and *DGAT*1 coexpression for TAG accumulation has been reported in tobacco [25]. Similarly, in sorghum, a TAG content of 3–8.4% of leaf DW has been reported for *WRI*1 coexpressed with *DGAT* and *OLE* [30]. These data support the conclusion that transcription factor *WRI*1 is a critical factor for the hyperaccumulation of TAG. However, the constitutive expression of multiple lipogenic factors can create a certain amount of toxicity to the cell, limiting tissue culture regeneration or compromising plant development and biomass accumulation [27].

The energycane lines P5, P8, and P16, which had TAG accumulations of 0.65%, 0.9%, and 1.52% of leaf DW and lacked *WRI*1 expression, did not display obvious growth retardation when compared to WT (Fig. 2F; Table 1), while sugarcane line 1565 with expression of *WRI*1 and TAG accumulation of 4.87% of leaf DW produced only 46% of the biomass of WT [28]. In addition, the tissue culture recalcitrance of energycane may create a bottleneck that leads to the selection of gene expression combinations with lower TAG accumulation levels. Future approaches should include strategies that avoid TAG accumulation in tissue culture or early plant development using stem-specific, inducible, or developmentally regulated promoters. Candidates for stem-specific promoters have been described [44, 45]. The transgenic energycane lines P5, P8, and P16 displayed a substantial increase in the accumulation of unsaturated fatty acids at the expense of saturated fatty acids (Fig. 4A, B). This indicates that the expression of *ZmDGAT*1-2 preferentially catalyzes the esterification of unsaturated fatty acids to diacylglycerol. This finding is consistent with earlier reports on sugarcane [10, 28] and *Arabidopsis* [20, 24].

Collectively, the optimization of expression cassettes using developmentally regulated or stem-specific regulatory elements, codon optimization, and stacking of additional lipid biosynthesis-related genes such as DOF4 [46] and a combination with CRISPR/Cas-mediated knockout of genes contributing to lipid hydrolysis could further elevate the accumulation of lipids in energycane.

## Conclusions

Energycane is a prime feedstock for the generation of renewable energy and bioproducts owing to its unsurpassed biomass yield and resilience under abiotic and biotic stress. In this work, a multigene expression/suppression strategy produced hyperaccumulation of TAG and total FA in leaves at levels exceeding non-modified energycane by 30- and almost twofold, respectively. Moreover, *ZmDGAT*1 expression and *SDP*1 suppression had the highest correlation with TAG accumulation. These results establish energycane as a promising production platform for lipids from vegetative biomass.

## Methods

### Construction of multigene expression vectors

Multigene expression and RNAi vectors were assembled using a conventional restriction enzyme digest of vector components and ligation, as described by Parajuli et al. [28]. In the multigene expression construct, *Zea mays* ubiquitin promoter (pZmUbi) and the terminator (tNos) of the nopaline synthase gene from *Agrobacterium tumefaciens* were used in the overexpression cassette of the *DGAT*1-2 gene from *Zea mays* (*ZmDGAT*1-2) (Fig. 1A); the ubiquitin promoter (pSbUbi) and the heat shock protein 18.2 terminator (tSbHSP) from *Sorghum bicolor* were used to drive the expression of *CysOLE*1 from *Sesamum indicum* (*SiCysOLE*1) (Fig. 1A). For the RNAi suppression of *TGD*1, an RNAi hairpin was custom synthesized (Genscript, Piscataway NJ) consisting of sense (244 bp) and anti-sense (244 bp) of *TGD*1 of *Saccharum* spp. hybrid separated by *Paspalum notatum*’s 4CL intron (94 bp). Similarly, the RNAi hairpin of *SDP*1 was custom synthesized (Genscript, Piscataway, NJ, USA) as the sense (278 bp) and anti-sense (278 bp) of *SDP*1 of *Saccharum* spp. hybrid separated with a 4CL intron (94 bp) from *P*. *notatum*. The hairpins of *TGD*1 and *SDP*1 were sub-cloned under transcriptional control of CaMV 35S promoter and terminator (Fig. 1B); the selectable marker gene *npt*II was placed under transcriptional control of the *Zea mays* ubiquitin promoter (pZmUbi) and the CaMV 35S terminator (t35S) (Fig. 1B).

### Tissue culture and genetic transformation of energycane

After six alternative genotypes were screened, energycane genotype CP 82–1592 [47] was selected for genetic transformation due to its efficiency in embryonic callus induction and plant regeneration. Immature leaf whorl cross-sections (Fig. 2A) were used as explants in the tissue culture, as described by Fouad et al. [37] to induce calli (Fig. 2B); linearized expression cassettes of transgenes were transformed into embryonic calli using the biolistic particle-delivery system; and putative transgenic plants were regenerated after selection with geneticin (Fig. 2C, D). The media used in callus induction, direct embryogenesis, transformation, selection, and shoot and root regeneration were prepared as described by Fouad et al. [37].

For the bombardment, the plasmid was prepared from an overnight culture of 5 mL *E*. *coli* strain TOP10 at 37 °C. The backbone of the plasmid was removed using restriction enzyme digestion with *Asc*I. The linearized fragment was gel extracted and purified as described by Fouad et al. [37]. A total of 13 shots of linearized fragment DNA were delivered in a 1:2 molar ratio (for *npt*II + tgdRNAi + sdpRNAi:DGAT + Ole) to callus using the Biolistic PDS-1000/He particle-delivery system (BioRad, Hercules, CA, USA) as described by Fouad et al. [37]. Rooted plantlets were transferred to a potting mix (Jolly Gardener C/G) after any media residues were washed from the roots and the plants had been covered for 5 days with a Magenta box to provide an environment with high humidity. In the growth chamber, the temperature was controlled at 25–28 °C during the day and 22–24 °C during the night. The photoperiod was set to 16 h light/8 h dark cycles with a light intensity of approximately 400 μmol m^−2^ s^−1^.

### NPTII immuno-chromatography assay

Leaf tissue from putative transgenic plants was ground in extraction buffer provided with the NPTII ImmunoStrip kit (Agdia Inc., Elkhart, IN, USA) and centrifuged at room temperature for 5 min at 16,100×*g*; the supernatant was transferred to a new microfuge tube, where it was absorbed by an ImmunoStrip. The presence of NptII was indicated by the development of two purple lines on the immuno-chromatography strip (control and test line), while non-transgenic control plants developed only one purple line (control line) (Fig. 2E).

### PCR analysis of genomic DNA extracts

Genomic DNA was extracted from young leaves of regenerated plants using the cetyl trimethyl ammonium bromide method [48]. Then, 100 ng genomic DNA was used as the template in a 20 µL PCR reaction. PCR amplification was conducted using Hot Start Taq DNA Polymerase (NEB; Ipswich, MA, USA) under the following conditions: 95 °C for 30 s, followed by 32 cycles of 95 °C for 30 s; 51.8 °C, 56.5 °C, 57.7 °C, or 58.2 °C for 30 s for *TGD*1, *npt*II, *CysOle*1, or *ZmDGAT*1-2, respectively; 68 °C for 1 min; and a final elongation at 68 °C for 5 min. The primers for each target gene are listed in Additional file 1: Table S1.

### Greenhouse propagation of transgenic, lipid-accumulating energycane

Transgenic energycane (Fig. 2F) was grown in a greenhouse and propagated by nodal stem cuttings to obtain biological replicates and three plants per transgenic line, and non-transgenic plants were each planted in a pot with a 15 cm diameter containing potting mix (Jolly Gardener C/G). Plants were grown under a drip fertigation system. In the greenhouse, the temperature was controlled by evaporation cooling to 25–29 °C during the day and 20–24 °C during the night, using the natural photoperiod with a maximum daily light intensity of approximately 500–1000 μmol m^−2^ s^−1^. The plants were irrigated and fertilized via drip fertigation. To compare the lipid gene expression and TAG accumulation between transgenic energycane and sugarcane, non-transgenic sugarcane CP 88–1762 and transgenic sugarcane line 1565, as described earlier by Parajuli et al. [28], were also grown in the same greenhouse in three biological replicates with similar lipid gene expression/suppression cassettes.

### Sampling of leaves for qRT-PCR, TAG, and FA analysis

The leaves of the transgenic lines and WT were numbered according to the system proposed by Kuijper et al. [49]. The top visible dewlap leaf blade of the plants that had been growing in the greenhouse for 1 month was used for these experiments. To ensure spatiotemporal correspondence between lipid gene transcripts and TAG and FA accumulation, the dewlap leaf was further divided into two halves at the midrib: one half was used for TAG and FA analyses, while the other half was used for qRT-PCR analysis. One sample was taken from each of the three plants, with a total of three biological replicates from each transgenic line. For the TAG and FA analyses, almost 100 mg fresh leaf tissue was needed for each sample. The samples were immediately freeze-dried in a lyophilizer (Labconco, Kansas, MO, USA) for 72 h. Samples containing lyophilized leaf tissue were delivered to Brookhaven National laboratory on dry ice and were immediately used for the TAG and FA analyses. For the qRT-PCR analysis, 0.1–0.2 g fresh leaf tissue was collected for each sample. The samples were flash-frozen in liquid nitrogen and were subsequently stored at − 80 °C before total RNA extraction.

### qRT-PCR analysis of lipid gene expression

Total RNA from leaf samples was extracted using TRIzol reagent (Ambion, Life Technologies, Thermo Fisher Scientific, Waltham, MA, USA); 1.0 µg total RNA from each sample was used for the cDNA synthesis using the High Capacity cDNA Reverse Transcription Kit (Applied Biosystems, Foster, CA, USA). The gene-specific primers described by Parajuli et al. [28] and shown in Additional file 1: Table S1 were used to evaluate the expression levels of the *ZmDGAT*1-2, *SiCysOLE*1, *TGD*1, and *SDP*1 genes. The *glyceraldehyde 3-phosphate dehydrogenase* (*GAPDH*) gene was used as a housekeeping gene for the normalization of transcripts [50]. qRT-PCR was conducted in a CFX Real-Time PCR detection system (Bio-Rad, Hercules, CA, USA) with SsoAdvanced Universal SYBR green supermix (Bio-Rad, Hercules, CA, USA) according to the conditions provided by Parajuli et al. [28]. The relative transcription levels of *ZmDGAT*1-2 and *SiCysOLE*1 were calculated using the 2^{Ct (GAPDH)–Ct (transgene)}^ method. The relative suppression levels of *TGD*1 and *SDP*1 were calculated using the 2−^ΔΔCt^ method [51].

### Analyses of TAG and total FA composition

TAG and FA analyses were carried out as described by Parajuli et al. [28]. In brief, 700 μL extraction solution in which methanol, chloroform, and formic acid were mixed at a ratio of 2:1:0.1 by volume was added to 10.0 mg lyophilized leaf tissue. After 3 h mixing on a vortex mixer, a hexane:diethyl ether:acetic acid solution (70:30:1 by volume) was added, and the total lipid extracts were divided for thin layer chromatography. Incubated in 1.0 mL boron trichloride-methanol at 80–85 °C for 40 min, TAG fractions were scraped from the plate under UV light and transmethylated into FA methyl esters (FAMEs). In the total FA analysis, after incubation in 1.0 mL boron trichloride-methanol, total lipid extracts were directly transmethylated into FAMES. FAMES were dissolved in 100.0 μL hexane and quantified via gas chromatography-mass spectrometry; 5.0 µg C17:0 was used as an internal standard.

### Statistical analysis

The data for qRT-PCR analysis, TAG content, and total FA content are expressed as means ± SDs. Statistical analysis was conducted by one-way ANOVA with the SPSS, version 20.0, program for Windows (SPSS Inc., https://www.ibm.com/analytics/data-science/predictive-analytics/spss-statisticalsoftware/). Values of *p* ≤ 0.05 were considered statistically significant. The Pearson’s correlation coefficient was evaluated using the Excel Analysis ToolPak (Microsoft, Redmond, WA, USA). Three independent biological replicates were used for each statistical analysis.

## Supplementary Information

**Additional file 1. Table S1**. List of primers used for gene expression analysis; **Table S2**. Summary of PCR analysis of transgenic lines; **Table S3**. Summary of TAG content and expression/suppression of (trans)genes in leaves of transgenic sugarcane; **Table S4**. Correlation of total FA content with TAG content in transgenic energycane; **Figure S1**. PCR analysis of transgenic plants.

## Data Availability

The datasets used and/or analyzed during the current study are available from the corresponding author upon reasonable request. Materials will be made available under a material transfer agreement.

## Abbreviations

*DGAT*1-2: *Diacylglycerol acyltransferase*1-2
DW: Dry weight
FA: Fatty acid
*GAPDH*: *Glyceraldehyde 3-phosphate dehydrogenase*
NPTII: Neomycin phosphotransferase II
*OLE*1: *Oleosin1*
qRT-PCR: Real-time quantitative reverse transcription PCR
*SDP*1: *SUGAR-DEPENDENT1*
*TGD*1: *Trigalactosyl diacylglycerol*1
TAG: Triacylglycerol
Ubi: Ubiquitin
*WRI*1: *WRINKLED*1
