# Supplementary material for: Metabolic engineering of energycane to hyperaccumulate lipids in vegetative biomass
Source: BMC Biotechnol. 2022 Aug 30;22:24. doi: 10.1186/s12896-022-00753-7 (PMC9425976; doi:10.1186/s12896-022-00753-7)
Supplement: Supplementary file 1 — Additional file 1. Table S1. List of primers used for gene expression analysis; Table S2. Summary of PCR analysis of transgenic lines; Table S3. Summary of TAG content and expression/suppression of (trans)genes in leaves of transgenic sugarcane; Table S4. Correlation of total FA content with TAG content in transgenic energycane; Figure S1. PCR analysis of transgenic plants. [file 12896_2022_753_MOESM1_ESM.docx]

**Supplementary Information**

**Additional file 1.** Table S1. List of primers used for gene expression analysis; Table S2. Summary of PCR analysis of transgenic lines; Table S3. Summary of TAG content and expression/suppression of (trans)genes in leaves of transgenic sugarcane; Table S4. Correlation of total FA content with TAG content in transgenic energycane; Figure S1. PCR analysis of transgenic plants

**Table S1 List of primers used for PCR and gene expression analysis**

| **Primer name** | **Sequence (5’ – 3’)** | **Amplicon length (bp)** | **Usage** |
| --- | --- | --- | --- |
| pZmUbi-F | ATCTCTGTCGCTGCCTCTG | 2,100 | PCR |
| ZmDGAT1-2-R | CCACAGGTGGAAGAAGCAGT |  |  |
| pSbUbi-F | ACGAACTCCACGACGACAC | 1,956 | PCR |
| CYSOLE1-R | GCTGAAACTGAAACCGGAGA |  |  |
| e35S_TGD1_Fw2 | TGACAAGCTGACTCTAGCAG | 995 | PCR |
| TGD1_R | AAACACTTGAGATTTCAGCA |  |  |
| NPTII-F | AGACAATCGGCTGCTCTGAT | 986 | PCR |
| 35S_NPTII-R | GGTAATGGGGGATCTGGATT |  |  |
| RT_GAPDH-F | CACGGCCACTGGAAGCA | 152 | qRT-PCR |
| RT_GAPDH-R | TCCTCAGGGTTCCTGATGCC |  |  |
| RT_ZmDGAT1-2-F | TACATCAACCCGATCGTGAA | 160 | qRT-PCR |
| RT_ZmDGAT1-2-R | CCACAGGTGGAAGAAGCAGT |  |  |
| RT_CYSOLE1-F | GCGGGCTCACAGACTAGC | 154 | qRT-PCR |
| RT_CYSOLE1-R | GCTGAAACTGAAACCGGAGA |  |  |
| RT_tgd1-F | CAGGTGTCGGAGCAGACC | 155 | qRT-PCR |
| RT_tgd1-R | CGAGGGACCACGAGGTAGT |  |  |
| RT_sdp1-F | TCGGCTTTGATAGGATAGGTG | 152 | qRT-PCR |
| RT_sdp1-R | TGAGATCCCTCAGAAGCCTTT |  |  |
| RT_NPTII-F | AGACAATCGGCTGCTCTGAT | 156 | qRT-PCR |
| RT_NPTII-R | CTGTGCTCGACGTTGTCACT |  |  |

Note: *DGAT*1-2: *Diacylglycerol acyltransferase1-2*; *GAPDH*: *Glyceraldehyde 3-phosphate dehydrogenase*; NPTII: Neomycin phosphotransferase II; *OLE*1: *Oleosin1*; *SDP*1: *SUGAR-DEPENDENT*1; *TGD*1: *Trigalactosyl diacylglycerol*1; Ubi: Ubiquitin; F/Fw: forward primer. R/Rv: reverse primer.

**Table S2 Summary of PCR analysis of transgenic lines**

| **Detected PCR amplicons** | **PCR positive lines** | **Lines selected for TAG and qPCR**  **analysis** |
| --- | --- | --- |
| *ZmDGAT*1-2/*SiCysOLE*1/*TGD*1/*NPT*II | 8 | P5; P8; P16; P24; P26; P27; P28 |
| Only *TGD1*/*NPT*II | 8 |  |
| Only *ZmDGAT*1-2/*SiCysOLE*1/*NPT*II | 5 | P6; P7; P20 |
| Only *ZmDGAT*1-2/*NPT*II | 5 | P23 |
| Only *SiCysOLE*1/*NPT*II | 4 |  |
| Only *NPT*II | 1 |  |
| **Total** | 31 |  |

**Table S3 Summary of TAG content and expression/suppression of (trans) genes in leaves of transgenic sugarcane**

| **Line** | **Leaf TAG**  **(% of DW)** | **Expression / Suppression of transgenes in leaf tissue** | | | | |
| --- | --- | --- | --- | --- | --- | --- |
|  |  | ***ZmDGAT*1-2** | ***SiCysOLE*1** | ***TGD*1 (%)** | ***SDP*1 (%)** | ***SbWRI*1** |
| CP 88-1762 | 0.03±0.01^a^ | 0.00±0.00^a^ | 0.00±0.00^a^ | 99±8^a^ | 100±9^a^ | 0.00±0.00^a^ |
| 1565 | 4.87±1.40^b^ | 0.19±0.02^d^ | 0.02±0.00^b^ | 114±5^a^ | 30±3^b^ | 0.05±0.00^b^ |

Note: TAG value represents the leaf samples from three biological replications. Values are means ± SD. Values within one column with different letters were significantly different at p ≤ 0.05 according to a one-way ANOVA test and the Duncan’s Multiple Range Test (MRT) post hoc test. Expression of transgenes is shown relative to *GAPDH*. Suppression of RNAi target genes is shown in percentage of that of control. The non-modified sugarcane cultivar CP 88-1762 was used as a negative control (WT).

**Table S4 Correlation of total FA content with TAG content in transgenic energycane**

|  | **Total FA** | **TAG** |
| --- | --- | --- |
| **Total FA** | 1 |  |
| **TAG** | 0.934^**^ | 1 |

Note: Highly significant differences in correlation were detected according to a one-way ANOVA test and the Duncan’s Multiple Range Test (MRT) post hoc test. * Correlation is significant at the 0.05 level (p ≤ 0.05); ** Correlation is very significant at the 0.01 level (p ≤ 0.01).

**Figure S1 PCR analysis of transgenic plants.**

**A** to **D**. The PCR amplification of *ZmDGAT*1-2, *SiCysOLE*1, *TGD*1, and *NPT*II, respectively. PC. Positive control (plasmid used for transformation), Genomic DNA extracts of energycane lines that tested positive in NPTII immuno-chromatography (P5; P8; P16) and non-transgenic plant (WT) used as negative control. Arrows indicate target amplicon.


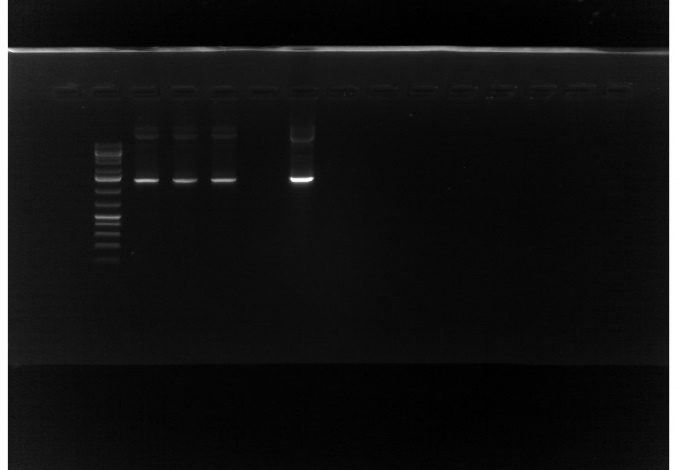


P5 P8 P16 WT PC


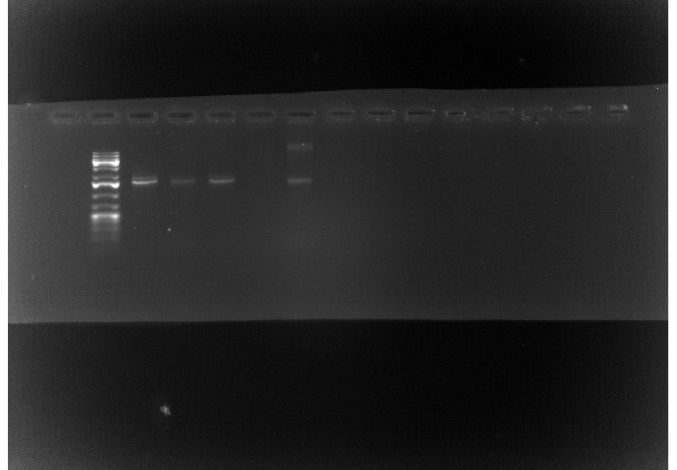


P5 P8 P16 WT PC


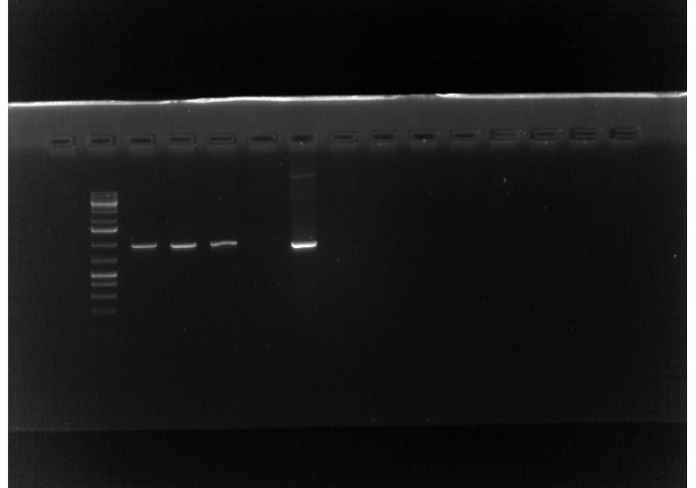


P5 P8 P16 WT PC


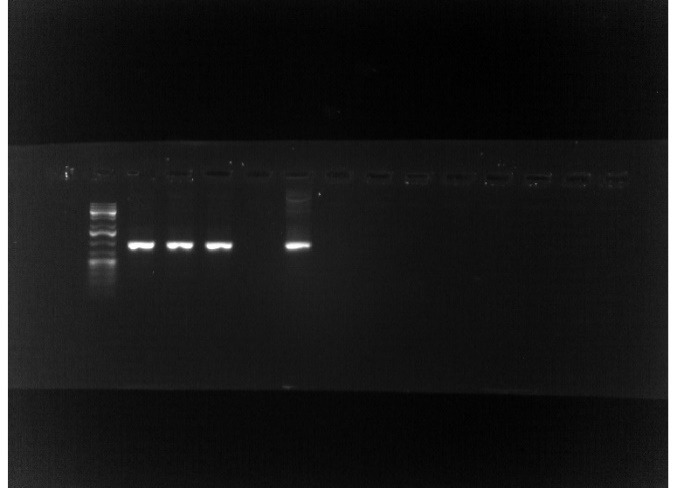


P5 P8 P16 WT PC

**(A)**

**(B)**

**(C)**

**(D)**
